# Zygomatic-maxillary cortical bone thickness in hyper, normo and hypodivergent patients

**DOI:** 10.1590/2177-6709.26.1.e211965.oar

**Published:** 2021-03-10

**Authors:** Julyano Vieira da COSTA, Adilson L. RAMOS, Liogi IWAKI

**Affiliations:** 1 Universidade Estadual de Maringá, Departamento de Odontologia (Maringá/PR, Brazil).

**Keywords:** Orthodontics, Orthodontic anchorage procedures, Cone beam computed tomography

## Abstract

**Objective:**

The aim of this study was to evaluate the thickness of the zygomatic-maxillary cortical bone using computed tomography in different skeletal patterns.

**Methods:**

A total of 54 patients of both sexes, divided into three groups according to the vertical skeletal pattern, were evaluated for cortical bone thickness of the anterior slope of the zygomatic process of the maxilla, using cone beam computed tomography. Measurements were made at 2mm, 4mm, 6mm, 8mm and 10mm above from first molar mesial root apex. Vertical skeletal pattern was determined by Frankfurt mandibular angle (FMA).

**Results:**

The hyperdivergent pattern had the lowest cortical thickness value, nevertheless, no patient in the hyperdivergent group presented cortical thickness exceeding 2mm, and no patient in the hypodivergent group presented cortical thickness less than 1mm. However, the correlation between cortical thickness and mandibular plane angle was weak and not significant.

**Conclusion:**

Although higher prevalence of thick cortical was observed in the hypodivergent patients, and thin cortical groups in the hyperdivergent group, the vertical skeletal pattern could not be used as determinant of the zygomatic-maxillary cortical thickness.

## RESUMO

**Objetivo:**

O objetivo do presente estudo foi avaliar a espessura da cortical óssea zigomático-maxilar por meio de tomografia computadorizada em diferentes padrões esqueléticos.

**Métodos:**

Um total de 54 pacientes de ambos os sexos, divididos em três grupos, de acordo com o padrão esquelético vertical, foi avaliado quanto à espessura da cortical óssea na vertente anterior do processo zigomático da maxila, por meio de tomografia computadorizada de feixe cônico. As medidas foram feitas a 2mm, 4mm, 6mm, 8mm e 10mm acima do ápice da raiz mesial do primeiro molar. O padrão esquelético vertical foi determinado pelo Ângulo do Plano Mandibular (FMA).

**Resultados:**

O padrão hiperdivergente apresentou o menor valor de espessura de cortical. No entanto, nenhum paciente do grupo hiperdivergente apresentou espessura cortical superior a 2mm e nenhum paciente do grupo hipodivergente apresentou espessura cortical inferior a 1mm. No entanto, a correlação entre a espessura da cortical e o ângulo do plano mandibular foi fraca e não significativa.

**Conclusão:**

Embora tenha havido uma maior prevalência de cortical espessa no grupo hipodivergente e cortical fina no grupo hiperdivergente, o padrão esquelético vertical não pode ser utilizado como determinante da espessura cortical zigomático-maxilar.

## INTRODUCTION

The use of miniplates and other temporary anchorage devices (TADs), have increased the possibilities of orthodontic movement, such as intrusion and distalization of anterior and posterior teeth.^1^ ^,^ ^2^

Some studies have demonstrated success in the treatment of patients considered borderline for the indication of orthognathic surgery, when treated with the aid of these devices. However, the stability of TADs depends on the quality and thickness of the cortical bone, which may be related to the skeletal pattern of the patient.^3^ ^,^ ^4^

Miniplate fixation is obtained by mechanical retention in the cortical bone, therefore, justifying the dependence on adequate bone thickness^5^. Studies have suggested that patients with a vertical growth pattern tend to present lower thickness values of the buccal and lingual bone plates at the level and above the apex of permanent teeth, when compared with patients with a horizontal growth pattern. However, there are few studies specifically evaluating the area of the zygomatic pillar.^4^ ^-^ ^7^

Cone beam computed tomography enables cortical bone thickness measurement in a real proportion, without presenting distortions and with a relatively lower dose of radiation, compared to traditional computed tomography. The imaging resource is fundamental for measuring the cortical thickness, especially in the zygomatic-maxillary region, which has been widely used for insertion of TAD devices.^8^ ^-^ ^10^

Thus, the aim of this study was to evaluate the zygomatic-maxillary cortical bone thickness in different vertical skeletal patterns, using cone beam computed tomography images.

## MATERIAL AND METHODS

The study sample consisted of volumetric computed tomography files of 54 patients (29 female and 25 male) from a database of tomography images belonging to a private Dental Radiology center (Maringá/PR, Brazil) and private clinic of professionals in the field of Dentistry in this same city.

The study was submitted to the Permanent Research Ethics Committee on Research Involving Human Beings (COPEP-UEM, *Universidade Estadual de Maringá)*, in accordance with the guidelines and regulatory rules on researches involving human beings (resolution nº. 196/96 of the National Council of Health), (CAAE #09159212.0.0000.0104). 

Complete eruption of the permanent teeth from the right second molar to the left second molar was an inclusion criteria. Women at the stage of menopause and patients with craniofacial anomalies were excluded from the study.

The sample calculation was made for a test power of 80% and level of significance of 5%, standard deviation of 0.45 and difference to be detected of 0.5mm.^11^ As a result, the number of 14 patients in each group was obtained. 

Tomographs were taken in i-CAT^®^ equipment (Imaging Sciences International, Hatfield, PA, USA) in single rotation (360º), 120 kvp, 23.87mAs and exposure time of 40 seconds. The protocol used was of the complete skull, with a 16*x*13 cm field of vision and voxel size of 0.3mm. The patients were oriented in a standardized position of the head, so that the Frankfurt plane would be set parallel to the ground, and the median sagittal plane, perpendicular to the ground. The images generated were saved in DICOM format (Digital Imaging and Communications in Medicine). Dolphin software^®^, version 11.7 Premium (Chatsworth, CA, USA) was used to evaluate the measurements.

For measurement procedure, the image was centralized on the axial slice, and the Frankfurt plane was positioned parallel to ground in sagittal slice. Anteroposterior cut was defined over the middle of the mesial-buccal root of the first permanent molar in each side. The measurements were taken on the coronal slice, with magnification of up to 200%, to facilitate visualization of the desired site, in a dark room, on a high-resolution monitor, by a single professional. 

**Figure 1:**
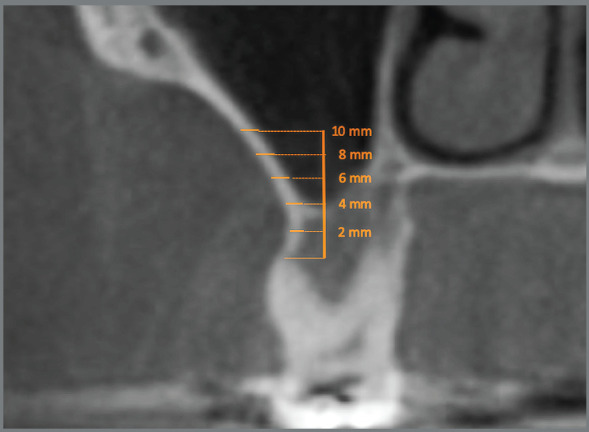
Reference lines used for the cortical bone thickness measurements.

A reference line perpendicular to Frankfurt plane, starting from the apex of the mesiobucal root of the maxillary first molars, was drawn. On this perpendicular line, references lines (parallel to Frankfurt plane) were drawn at 2mm, 4mm, 6mm, 8mm and 10mm from the apex of the molars. Cortical bone thickness was evaluated in the intersection of this line to the anterior slope of zygomatic-maxillary bone, on both right and left sides of the maxilla (Fig 1).^10^ ^,^ ^12^ ^,^ ^13^ ^,^ ^14^

Measurement of the skeletal growth pattern was made on the lateral images (from the tomography), using the cephalometric variable FMA (Frankfurt Mandibular Plane Angle). Therefore, subjects with an angle between 21º and 29º were classified as normodivergent; those with an angle smaller than 21º or larger than 29º, were classified as hypodivergent and hyperdivergent, respectively.^4^ ^,^ ^15^

The sample was then divided into three groups: Group 1) Normodivergent (n = 23) (mean: 44.57 years; S.D.: 13.64); FMA (mean: 24.85; S.D: 2.58). Group 2) hypodivergent (n = 12) (mean: 44.75 years; S.D.: 15.09); FMA (mean: 16.05; S.D: 3.08) and Group 3) hyperdivergent (n = 19) (mean: 40.37 years; S.D.: 14.46); FMA (mean: 34.22; S.D: 4.66). 

The landmarks, lines and planes were established by a single operator. Twenty days after the first measurement, 13 images were traced again, to determine the reliability of the data. In a similar manner, 40 days after the initial stage, 13 images were drawn again, following the same references, and measurements of the buccal cortical bone thickness of the maxilla were taken.

## STATISTICAL ANALYSIS

The Kolmogorov-Smirnov test was used to verify the sample distribution of the variables. In view of the normality of the data (*p*< 0.05), the *t*-test was used for comparison between the sides, and between the skeletal pattern and gender. ANOVA test was used for comparison between the groups.

The Pearson correlation test was applied to the cortical bone thickness *versus* skeletal growth pattern (FMA).

All statistical tests were performed with the Statistical Program (version 7.0; StatSoft Inc., Tulsa, OK, USA), adopting the level of significance for *p*< 0.05.

## RESULTS

The results of systematic and random errors demonstrated reduced values (0,38 to 0.63), that were not significant.

The *t*-test for dependent samples showed no significant difference between the cortical bone thickness values between the right and left sides (*p*< 0.05) (Table 1). There were also no significant differences between the sexes within each group, or between the groups (Table 2). For these reasons, the groups were treated by using the means without distinction between sides and sex. 

**Table 1:** Comparison among the mean cortical thickness values evaluated along the vestibular zygomatic-maxillary slope, by using the t-test for dependent samples, between the right and left sides.

	Right Side		Left Side		P
	Mean	S.D.	Mean	S.D.	
HYPERDIVERGENT					
2mm	1.44	0.44	1.40	0.46	0.80
4mm	1.30	0.39	1.28	0.32	0.86
6mm	1.19	0.32	1.23	0.35	0.71
8mm	1.19	0.26	1.29	0.34	0.28
10mm	1.22	0.24	1.27	0.40	0.64
Mean	1.40	0.29	1.39	0.30	0.91
NORMODIVERGENT					
2mm	1.85	0.78	1.69	0.49	0.35
4mm	1.67	0.75	1.46	0.37	0.19
6mm	1.50	0.58	1.43	0.38	0.58
8mm	1.37	0.49	1.53	0.64	0.29
10mm	1.36	0.49	1.53	0.61	0.24
Mean	1.67	0.56	1.63	0.38	0.74
HYPODIVERGENT					
2mm	1.65	0.59	1.47	0.36	0.37
4mm	1.45	0.53	1.31	0.3	0.44
6mm	1.43	0.57	1.24	0.32	0.30
8mm	1.37	0.45	1.29	0.49	0.65
10mm	1.48	0.58	1.30	0.46	0.40
Mean	1.57	0.51	1.42	0.38	0.39

**Table 2:** Comparison among the mean cortical thickness values evaluated along the vestibular zygomatic-maxillary slope, by using the *t*-test for dependent samples, between the skeletal pattern and sex.

	Male	Female	p
	X (S.D.)	X (S.D.)	
HYPODIVERGENT	1.50 (0.42)	1.35 (0.32)	0.46
NORMODIVERGENT	1.70 (0.50)	1.69 (0.36)	0.26
HYPERDIVERGENT	1.47 (0.14)	1.40 (0.27)	0.81

There was statistically significant difference between the mean values of buccal cortical bone thickness between the hyperdivergent and normodivergent patients, in the areas closer to the root apex (at 2mm, 4mm and 6mm). However, at 8mm and 10mm, there was no difference between groups (Table 3). Furthermore, Pearson correlation test between the buccal cortical bone thicknesses and the skeletal growth pattern (FMA) presented low values, that were not significant (Table 4).

**Table 3:** Comparison among the mean cortical thickness values evaluated along the vestibular zygomatic-maxillary slope, between the groups, by using one-way ANOVA.

	HYPERDIVERGENT (n=19)	NORMODIVERGENT (n=23)	HYPODIVERGENT (n=12)	*p*
2mm	1.42 (0.44)^A^	1.77 (0.65)^B^	1.56 (0.49)^AB^	0.01*
4mm	1.29 (0.35)^A^	1.57 (0.59)^B^	1.38 (0.44)^AB^	0.02*
6mm	1.21 (0.33)^A^	1.46 (0.49)^B^	1.34 (0.46)^AB^	0.02*
8mm	1.24 (0.30)^A^	1.45 (0.57)^A^	1.33 (0.46)^A^	0.09
10mm	1.24 (0.33)^A^	1.44 (0.56)^A^	1.39 (0.52)^A^	0.13
Média	1.39 (0.29)^A^	1.65 (0.47)^B^	1.50 (0.45)^AB^	0.01*

**Table 4:** Correlation between FMA angle and the cortical bone thickness.

Correlation	r	*p*
FMA x 2mm	-0.16	0.07
FMA x 4mm	-0.12	0.17
FMA x 6mm	-0.14	0.11
FMA x 8mm	-0.08	0.32
FMA x 10mm	-0.11	0.22
FMA x Mean	-0.15	0.09

## DISCUSSION

Temporary anchorage devices have allowed tooth movements in patients considered borderline cases for orthognathic surgery. However, skeletal anchorage may not be stable, and this fact could be related to the cortical bone thickness, which differs among patients.^3^ ^,^ ^4^ In this context, more studies about zigomatic cortical thickness are being encouraged, and the present study aimed at clarifying the anatomic variability of these areas.^4^ ^,^ ^6^ ^,^ ^16^

Recently, the importance of cortical thickness and bone density for the insertion of temporary anchorage in the infrazygomatic crest region and the mandibular ramus was reported, relating to possible failure of these devices.^13^ ^,^ ^16^

The hyperdivergent pattern may present thin cortical thickness values, as previously reported.^6^ In fact, the buccal and lingual cortical bone in hypodivergent patients (ranging from 1.0mm to 2.6m) was thicker in comparison with that of hyperdivergent patients (ranging from 0.08 to 0.64mm) in the present study, however with no statistical significance.^4^ ^,^ ^15^

A tendency for thicker than 2.5mm cortical bone was observed in dry skulls mandibles from Japanese and Indian subjects.^6^ In the present study, this trend was observed; however, closer to the maxillary sinus (at 8 mm and 10 mm), the mean difference among the facial patterns was not significant (Table 3).

Horner et al.^4^ reported that hyperdivergent patients presented minimum cortical bone thickness values between 0.6 and 0.7mm, which were similar to those in the present study for subjects with dolicofacial pattern. These dimensions may represent a problem for the stability of screws in this area, considering that at least 1 mm thickness of cortical bone would be adequate.^15^ For this reason, miniplate screws of increased diameter (between 2.3 and 2.5 mm) are used to overcome this limitation. Even so, in average a smaller area of cortical contact may be expected in hyperdivergent patients than in patients with other growth patterns. Hypodivergent and normodivergent patients in the present study presented minimum values of 1 and 0.9 mm respectively, limiting them to a lesser extent with regard to this requisite. Moreover, in subjects with a normodivergent and hypodivergent pattern, measurements of up to 3.4 mm thickness were found, and although the mean values of the groups did not differ significantly, there was a higher proportion of cases with thicker cortical among the hypodivergent patients. 

**Figure 2:**
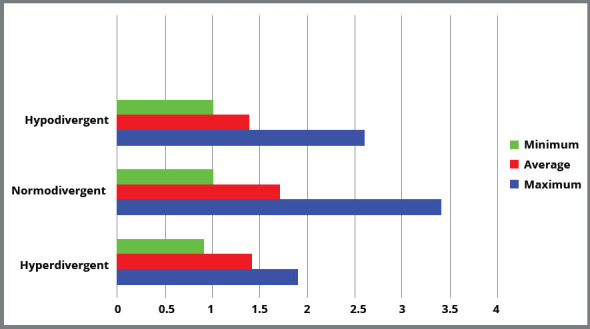
Comparative graph of measuring the cortical bone thickness along the vestibular zygomatic-maxillary slope among the hypodivergent, normodivergent and hyperdivergent cephalic patterns.

No patient in the hyperdivergent group presented a thickness greater than 2 mm; and in the same way, no patient in the hypodivergent group presented a cortical thickness of less than 1 mm (Fig 2). It is worth emphasizing that these differences in behavior of the cortical bone were more evident up to distance of 6 mm above the root apex of the permanent maxillary first molars (Table 3). It is expected that higher insertion of the TAD relates to lower thickness of cortical bone for all the skeletal patterns.^16^ ^-^ ^18^

The results demonstrate that there was no significant correlation between zygomatic cortical bone thickness and skeletal pattern (FMA). Therefore, an individualized evaluation would be necessary, since one hyperdivergent patient may have a thick or a thin cortical bone, as well as a hypodivergent patient may have either thick or thin cortical thickness for TAD insertion.

## CONCLUSION

Although there was higher prevalence of thick cortical in hypodivergent patients, and thin cortical in hyperdivergent group, skeletal pattern (FMA) could not be used as predictor for zygomatic-maxillary cortical bone thickness.
